# Comparison the Marginal and Internal Fit of Metal Copings Cast from Wax Patterns Fabricated by CAD/CAM and Conventional Wax up Techniques

**Published:** 2013-09

**Authors:** M Vojdani, K Torabi, E Farjood, AAR Khaledi

**Affiliations:** a Dept. of Prosthodontics, School of Dentistry, Shiraz University of Medical Sciences, Shiraz, Iran.; b Postgraduate Student, Dept. of Prosthodontics, School of Dentistry, Shiraz University of Medical Sciences, Shiraz, Iran.

**Keywords:** Marginal Fit, Internal Fit, Wax Patterns, Metal-Ceramic Crowns, CAD/CAM

## Abstract

**Statement of Problem: **Metal-ceramic crowns are most commonly used as the complete coverage restorations in clinical daily use. Disadvantages of conventional hand-made wax-patterns introduce some alternative ways by means of CAD/CAM technologies.

**Purpose:** This study compares the marginal and internal fit of copings cast from CAD/CAM and conventional fabricated wax-patterns.

**Materials and Method:** Twenty-four standardized brass dies were prepared and randomly divided into 2 groups according to the wax-patterns fabrication method (CAD/CAM technique and conventional method) (n=12). All the wax-patterns were fabricated in a standard fashion by means of contour, thickness and internal relief (M1-M12: representative of CAD/CAM group, C1-C12: representative of conventional group). CAD/CAM milling machine (Cori TEC 340i; imes-icore GmbH, Eiterfeld, Germany) was used to fabricate the CAD/CAM group wax-patterns. The copings cast from 24 wax-patterns were cemented to the corresponding dies. For all the coping-die assemblies cross-sectional technique was used to evaluate the marginal and internal fit at 15 points. The Student’s t*-* test was used for statistical analysis (α=0.05).

**Results: **The overall mean (SD) for absolute marginal discrepancy (AMD) was 254.46 (25.10) um for CAD/CAM group and 88.08(10.67) um for conventional group (control). The overall mean of internal gap total (IGT) was 110.77(5.92) um for CAD/CAM group and 76.90 (10.17) um for conventional group. The Student’s t-test revealed significant differences between 2 groups. Marginal and internal gaps were found to be significantly higher at all measured areas in CAD/CAM group than conventional group (*p*< 0.001).

**Conclusion:** Within limitations of this study, conventional method of wax-pattern fabrication produced copings with significantly better marginal and internal fit than CAD/CAM (machine-milled) technique. All the factors for 2 groups were standardized except wax pattern fabrication technique, therefore, only the conventional group results in copings with clinically acceptable margins of less than 120um.

## Introduction

Metal ceramic are still the most widely used material for fabricating complete coverage crowns and fixed partial dentures [1], and is considered as the standard treatment in dentistry. The traditional technique for fabricating the metal substructure is the lost-wax technique and using various metal alloys for casting [2].

Conventionally, wax patterns were fabricated with wax and waxing instruments for example the popular PKT instruments. Wax is used to make the patterns because it can be conveniently manipulated, precisely shaped and can also be completely eliminated from the mold by heating [3]. The fabrication of the wax pattern is the most critical and labor-intensive step in making the porcelain fused-metal crown. In this time-consuming task, the wax–up’s quality is dependent on the skilled labor of the individual [4]. Zeltser et al. [5] found that the act of removing a wax pattern from a die with a shoulder margin causes an average of 35μm opening of the margin before investing. Because of the wax pattern’s color and glossy surface, small defects can be difficult to identity [3]. Wax has several inherent limitations namely, delicacy, thermal sensitivity, elastic memory and a high coefficient of thermal expansion (CTE) [6].

Today, by introducing different CAD/CAM systems, it is possible to fabricate the wax patterns made from castable materials and omit several limitation of conventional wax-up technique. CORiTEC (imes-icore GmbH; Eiterfeld, Germany), Precident DCS system (DSC-dental; Allschwill, Switzerland) and Everest system (Kavo-Everest; Leutkirch, Germany) are among the CAD/CAM systems that have the ability to mill frameworks made from castable acrylic [7]. Using CAD/ CAM systems have many advantages such as producing higher and more uniform-quality restorations by using commercially formed blocks of material, standardizing restoration shaping processes and reducing production costs, labor and time [8]. Another advantage is the potential to enhance accuracy as they omit several fabrication steps used as waxing, investing and casting [6]. Given the simplicity of automatic margin detection and restoration design compared to manual waxing, it makes the most sense to use the CAD/CAM features where possible [9]. CAD/CAM systems also have some disadvantages. For example, the scanning systems have the limitation of finite resolution, which can result in edges that are slightly rounded. The point clouds obtained in scanning are transformed through a CAD software algorithm into a smooth and continuous surface, which can also lead to some internal inaccuracies. This can lead to interfering contacts at the incisal/occlusal edges and can be detrimental if they occur at the margins [6]. 

The fit of any restoration is determined by its marginal and internal fit [6]. A good marginal fit seems to be one of the most important technical factors for the long-term success of metal-ceramic crowns [10]. Increased marginal discrepancies expose the luting material to the oral environment, thus leading to cement dissolution and microleakage. The cement seal becomes weak, permits the percolation of bacteria, and can cause inflammation of the vital pulp. The *in-vivo* studies have provided evidence that a large marginal discrepancy in a fixed restoration correlates with a higher plaque index and reduced periodontal conditions [11]. Minimal marginal gaps results in less gingival irritation cement dissolution, recurrent caries and marginal discoloration [12-13]. The internal gap was defined as the perpendicular distance between the framework and the abutment teeth and it is the misfit of the coping at the occlusal/incisal and axial surfaces [14-15]. 

The internal fit should be uniform to avoid compromising either the retention or the resistance of the crown and should also provide an appropriate luting space [16]. An increase in cement thickness can lead to higher amounts of water absorption. Water absorption results in hydrolytic degradation of resin cements, thus reducing the elastic modulus and the mechanical properties [17-19]. Rekow et al. demonstrated that an excessively thick cement layer may cause residual stresses on the tensile surface as a result of the viscoelastic deformation of the cement material under cyclic loading. These increased tensile stresses may damage the veneering porcelain and initiate chipping of the veneering layer [20]. Apart from the mechanical properties of the material used, the internal fit also has a practical aspect. If too much space is lost as a rsult of large occlusal discrepancies, the intercuspal clearance available for veneering is reduced [21].

Many authors compare the marginal or internal fit of CAD/CAM fabricated restorations with conventional made metal copings or crowns [9, 14, 21-28]. However, it is difficult to compare these studies, because of variations in the sample size, measurements per specimens, measurement method, cement space and the CAD/CAM systems used. Several studies reported larger marginal or internal gap for CAD/CAM or CAM ceramic reconstruction than conventional PFM crowns [9, 14, 21-24]. CAD/CAM fabricated restorations also had better marginal fit than conventional cast restorations in several

studies [25-28].

There is no consensus on an acceptable maximum clinical marginal gap width. The values reported in the literature range from 50 to 200µm, suggesting the absence of an objective limit based on scientific evidence [29]. After the assessment of subgingival and supragingival margins, Christensen [30] reported that marginal discrepancies of 39μm or more, in visually accessible surfaces, are unacceptable. McLean and von Fraunhofer [31] reported that marginal gaps and, therefore cement film thickness, could range up to 120μm after cementation and be clinically acceptable. Theoretically, the internal space necessary for the cement is 20 to 40μm. As reported by Fransson et al. [32] the cement films in the range of 25-50um are seldom to be obtained; therefore the practical range for clinical acceptability of internal fit seems to be approximately 50 to 100um [33].

There are several basic methods for measuring marginal and internal gaps:

1. Direct view (External microscopic examination)
2. Cross-sectional technique after cementation and embedding (internal microscopic examination)
3. Impression technique (internal replica approach)
4. Weighing the light-body addition silicon 5) Explorer and visual examination [6, 34].

According to the advantages and disadvantages of both conventional wax up technique and CAD/CAM systems, and on the other hand the importance of marginal and internal fit, this hypothesis evolved that if using each of these method for fabricating the wax pattern have any significant influence on the marginal and internal fit of the final metal coping. 

The purpose of this study was to evaluate the marginal and internal fit of metal coping cast from wax patterns fabricated with two different fabrication techniques:

1. Milling from a solid acrylic resin block and
2. Conventional wax-up technique.

The null hypothesis was that no differences would be found in marginal and internal fit of metal coping cast from wax patterns fabricated from these two various methods. 

## Materials and Method

The cross-sectional (internal microscope) technique was used to evaluate the marginal and internal discrepancy of the models, by 230 magnifications.

**Experimental Models Fabrication**

**Figure 1:**
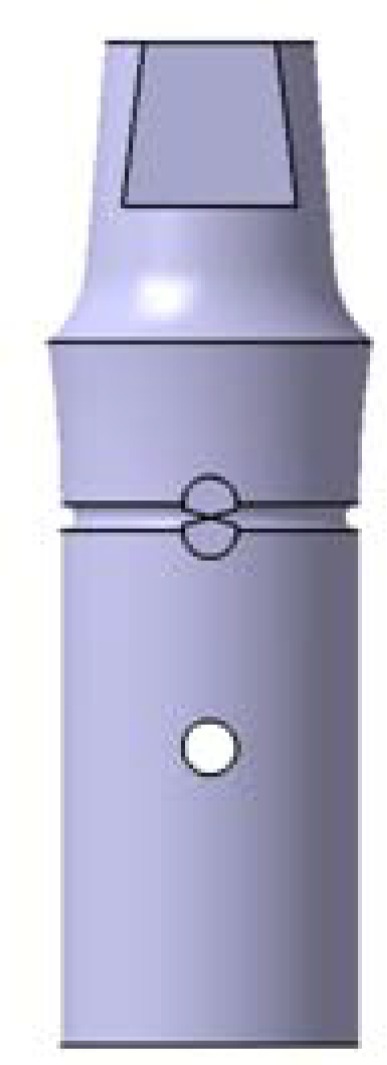
Virtual designed die

Twenty-four machined brass dies models were designed and prepared in a lathe (CNC350; Arix Co. Tainan Hesin, Taiwan) to simulate full coverage PFM crown preparation with a chamfer margin around the entire circumference. Preparation were standardized with a height of 5.5 mm, width of 6 mm at the margin, the convergence angle of 6 degrees, 0.7 mm chamfer width, chamfer radius of 2mm (R=2), and an anti rotational surface (Figure 1). To avoid any possible variations during the impression and casting stages, the brass dies were used as definitive dies to fabricate the restorations [11, 24-25, 28-29, 32, 35]. According to the method of wax pattern fabrication, they were randomly divided into one experimental group (CAD/CAM group) and one control group (conventional group) (n=12) and then numbered as M1 to M12 and C1 to C12 respectively. 

**Fabrication of wax-patterns**

Twelve standardized wax patterns were fabricated on brass dies for each group.

**Figure 2:**
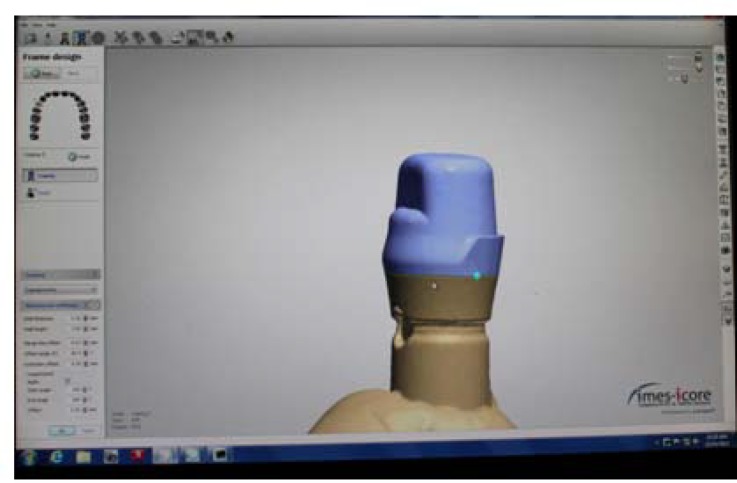
Designed coping by CAD system for CAD/CAM group and master wax-up

The CAD/CAM wax-patterns were produced with a milling machine(CORiTEC 340i; imes-icore GmbH, Eiterfeld, Germany) using a laser scanner (3Shape D810; 3Shape, Copenhagen K, Denmark) to digitize the dies, after they were sprayed with scan spray (Arti-Scan CAD/CAM Spray, Bausch Gmbh & Co. KG, Köln, Germany). The data were then transmitted to a software program (3Shape's CAD Design software, 3Shape, Copenhagen K, Denmark) in which the wax patterns were designed with a thickness of 0.5mm and 2mm lingual collar height. The appropriate emergence profile was selected to follow the emergence profile of die. Simulated die spacer was set at 30μm, starting 1mm from the margin [35-37] (Figure 2). Once the milling paths were computed by CAM system (CORiTEC iCAM V4, imes-icore GmbH) all the wax patterns were milled from a PMMA disc (CORiTEC PMMA Disc burn-out, imes-icore GmbH). The size of the smallest milling bur for PMMA was 1.0 mm.

For the conventional Group wax patterns fabrication, two coats of die spacer (PICO-FIT red; Renfert GmbH, Hilzingen, Germany) were applied 1mm away from the margin with a brush system. The bottles were kept closed between applications, and the brush was cleaned frequently with a thinner agent. According to the product catalogue, each layer of die spacer has a thickness between 12-15μm (approximately equal to 30um by 2 application, for standardization between 2 groups). After drying, a thin layer of separating medium (Picosep; Renfert GmbH, Hilzingen, Germany) was also applied to the dies.

**Figure 3:**
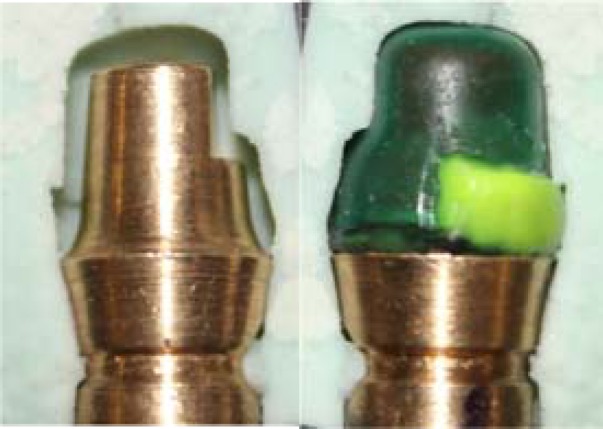
An index made from master wax-up, used to standardize the conventional group wax patterns’ contour and thickness

To standardize the contour and thickness of wax patterns fabricated with both methods, a master wax up was made on a spare die by the CAD data as for the CAD/CAM group. After casting and cementation on the die, a silicon index was made and cut into two pieces. The index was stabilized on the die by a groove and notches designed inferior to the margin (Figure 3).

**Figure 4:**
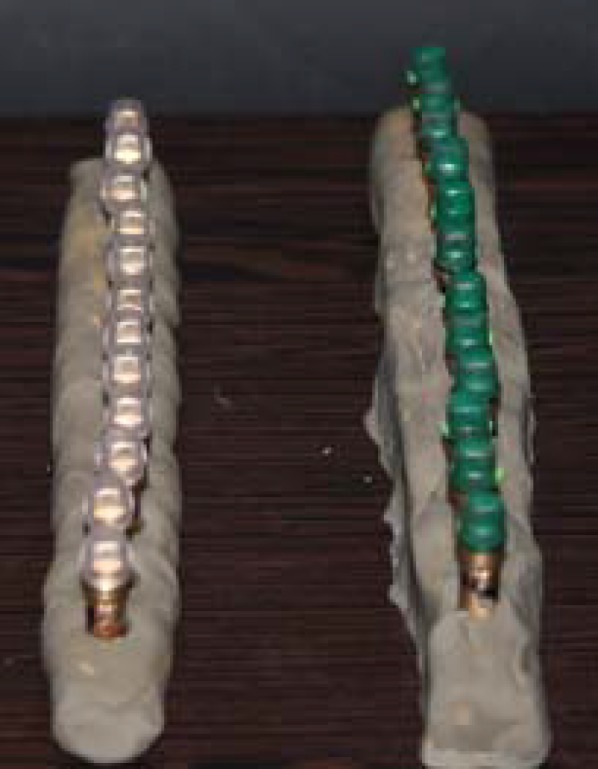
CAD/CAM and conventional groups were waxed (M1-M12 , C1-C12 respectively

Dip wax technique (Renfert Hotty; Renfert Gm-GmbH) was used to form wax copings (GEO-Dip; Renfert GmbH). Inlay wax (GEO classic; Renfert GmbH) was added and shaped by electric waxing instruments (Waxlectric II, Renfert GmbH) to form 2mm height lingual collar and to follow the emergence profile of the die, as dictated grossly by the index. The thickness of the copings was confirmed with a thickness gauge (POCO 2N; Kroeplin, Schluchtern, Germany) to be 0.5mm except the lingual collar. Finally to re-adapt the margin, the pattern was reflowed completely through the wax over a band approximately 1mm wide with a well-heated instrument, PKT No.1 (PKT waxing1; Dental USA lnc, McHenry, USA). Wax was then added to fill the depression, and when the pattern had cooled, the marginal excess was carved and the margin was burnished (Figure 4).

**Investing and casting**

Wax sprues were attached, and all twenty-four wax patterns (M1-M12 and C1-C12) were invested in a phosphate-bonded investment (Z4-C&B investment; Neirynck & Vogt,Schelle,Belgium),at a 24ml/100g (water/powder) mixing ratio. After heating to 950˚C for wax elimination (Preheating furnace Magma; Renfert GmbH, Hilzingen, Germany) the copings were cast in Ni-Cr Alloy (4all; Ivocolar vivadent, Schann, Liechtenstein) using a casting machine (Nautilus CC plus; Bego, Bremen,Germany). The castings (M1-M12 and C1-C12 according to their wax pattern code) were removed from the investment and cleaned with 50μm aluminum oxide particles (Basic master; Renfert GmbH, Hilzingen, Germany) at a pressure of 0.3MPa. The casting sprues were removed by using a polisher and grinder machine (DEMCO E96; CMP Industries LLC, Albany, NY) with a separating disc (Dentaurum J.P.; Winkelstroeter KG, Pforzheim, Germany).

The internal surfaces of the copings were examined using a binocular loupes (HEINE HR-C 2.5x, HEINE, Herrsching, Germany) and any visible metal nodules were removed with a tungsten carbide bur (No. H71EF; Brasseler GmbH and Go KG) with a handpiece (KaVo K9; KaVo dental GmbH,Biberach, Germany). To detect the invisible nodules or irregularities, the internal surfaces of the copings was checked on the master dies using vinyl poly-siloxane disclosing paste (Fit checker; GC Corporation). After removing the crown from the die, the contact spot, marked by the indicator on the inside of the copings was examined visually using a binocular loupes (HEINE HR-C 2.5x, HEINE, Herrsching, Germany), these marked spots were also removed with the same mentioned way, until no internal binding was occurred and a uniform thickness of disclosing paste achieved. Die spacers on the conventional group were cleaned with a steam cleaner (Vap-6; Zhermack technical, Badia Polesine, Italy).

**Cementation**

**Figure 5:**
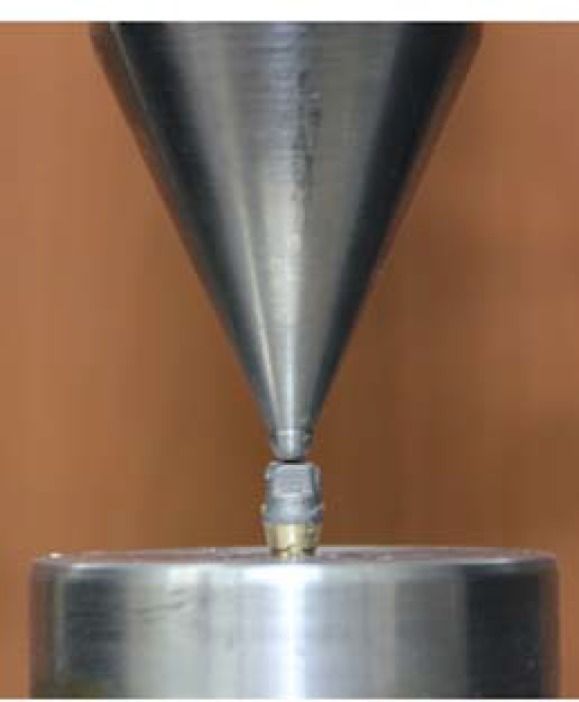
Cementation of the specimen with universal testing machine

Copings were cemented on their dedicated master dies with glass-ionomer cement (Ketac Cem EasyMix; 3M ESPE AG, Seefeld, Germany) by rocking motion and after removing the excess cement with a gauze pad, they were immediately placed in a constant load with universal testing machine (Zwick-Roell Z020; Zwick Gmb H & Co. KG, Ulm, Germany) at 50N [22, 37-38] for 10 minutes (Figure 5). The use of a loading apparatus would provide a more uniform load on all the specimens that standardized the cementation process for all models.

**Fixing and cutting the models**

**Figure 6:**
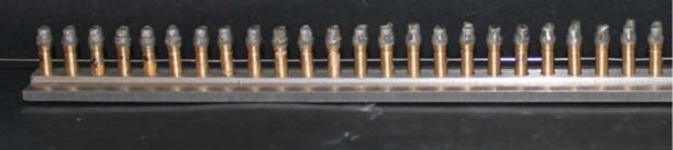
The specimens were aligned in a standard fashion after fixing on a machine-milled plate

**Figure 7:**
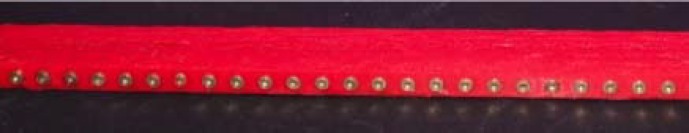
All the specimens were embedded in auto-polymerizing acrylic resin

The coping-die assemblies were fixed randomly in a straight line on a custom milled plate by means of screws, designed at lower surfaces of the dies. All the anti-rotational surfaces of the dies were aligned in one direction by crossing an orthodontic wire from the holes designed in the body of the dies (Figure 6). All the specimens were embedded in auto-polymerizing acrylic resin (DuraLay; Reliance Dental Co., Place Worth, IL) after vertical walls were placed around the fixed specimens (Figure 7).

**Figure 8:**
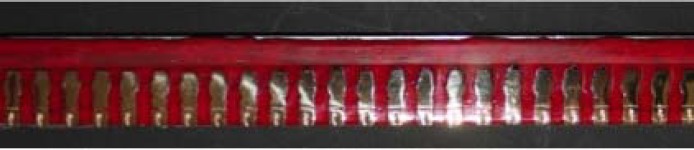
Polished sectioned resin block

**Figure 9:**
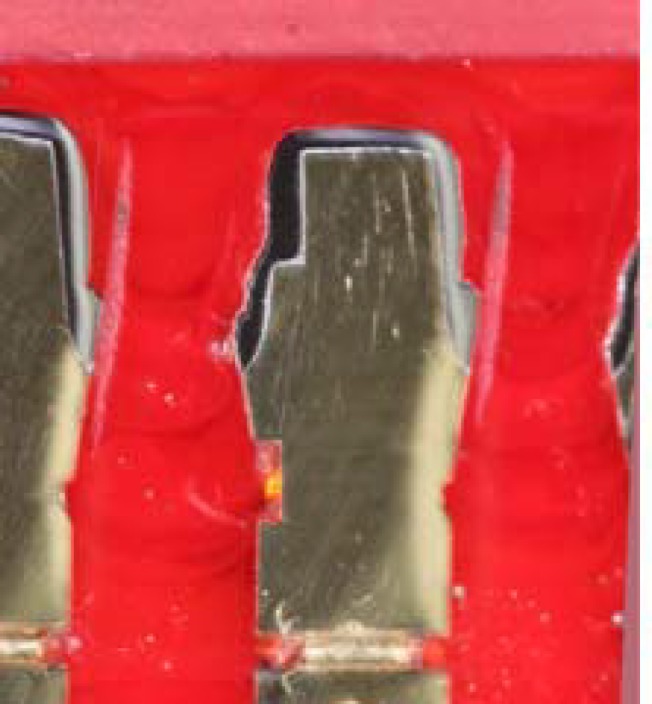
Close-up view of sectioned block

The Resin block was sectioned longitudinally into two halves, with a water jet cutter (Waterjet Cutting System; Jet Edge World Headquarters, St. Michael, USA). The left half --sectioned surfaces were polished by consecutive grinding with #400, #800, #1000, and #2000 silicon carbide papers (Silicon Carbide Paper MATADOR waterproof; Germany) (Figure 8 and 9).

**Measurement of marginal and internal fit**

Holmes et al [39] suggested some terms to be used in measuring the marginal and internal fit; such as absolute marginal discrepancy, marginal gap, and internal fit at axial and occlusal surfaces. These terminologies were used to measure the marginal and internal fit. To standard the measurement at axial surfaces of all specimens; three fine-longitudinal line were drawn on the acrylic resin between the sectioned copings by a fine indelible marking pen.

The sectioned resin block placed under a digital microscope (AM413FIT Dino-Lite Pro; Dino-Lite, Taipei, Taiwan) mounted on desktop stand (MS35B, Dino-Lite, Taipei, Taiwan) and connected to a personal computer (PC) via USB 2.0 connection and photographs were taken sequentially at 230x magnification, to measure the marginal and internal fit of the copings.

The following sites were evaluated:

1. Absolute marginal discrepancy at left and right side (AMD_L,R_): measured from the margin of the casting to the cavosurface angle of the die.
2. Marginal gap at left and right side (MG_L,R_): The perpendicular distance from the internal surface of the casting to the axial wall of the die at the margin.
3. Internal fit at axial walls (IGA_1-8_): the perpendicular distance from the internal surface of the casting to the axial wall of the die at the middle of chamfer margin at both sides and at 6 points defined by the lines that had been drown on the resin block.
4. Internal fit at occlusal wall (IGO_1-3_): the perpendicular distance from the internal surface of the casting to the axial wall of the die at left, right and middle of occlusal surface.

The camera produced an image of the site on computer monitor. The discrepancies were measured by placing the mouse pointer on the both surfaces. The software has the ability to show the perpendicular distance by two parallel lines that must place on the two surfaces. The distance was counted in microns on the screen. A digital image was made of a cell counting chamber (Neubauer Improved; Laboroptik GmbH, Friedrichsdorf, Germany) at the same magnification for use in calibration of the measurement software. To ensure that the software was correctly calibrated for the data collection, a periodic measurement of a known distance was made. Using this technique, a total of 15 single measurements were made around each specimen.

The means and standard deviations of the measurements per group were used for statistical analysis (SPSS 16.00 for windows; SPSS inc, Chicago, USA). The results of the 2 groups (n=12) were also compared using Student’s *t-* test (α=0.05).

## Results

The means and standard deviations for absolute marginal discrepancy (AMD), marginal gap (MG), axial internal gap (IGA), occlusal internal gap (IGO) and total internal gap (IGT) of both groups are summarized in table 1.

**Table 1:** Mean±SD value of marginal and internal gaps depending on fabrication method (independent t-tests) AMD: absolute marginal discrepancy, MG: marginal gap, IGA: internal gap axial, IGO: internal gap occlusal, IGT: internal gap total (Significant difference *p*< 0.05

	**Groups (mean±SD)**				
	**CAD/CAM**	**Conventional**	**t**	**df**	***p***
AMD	254.46±25.10	88.08±10.67	-21.13	22	<0.001
MG	157.37±20.63	69.54±15.60	-11.76	22	<0.001
IGA	81.41± 6.69	60.84± 6.05	-8.21	22	<0.001
IGO	189.08±16.16	119.72±24.40	-7.90	22	<0.001
IGT	110.77±5.92	76.90±10.17	-9.97	22	<0.001

**Figure 10:**
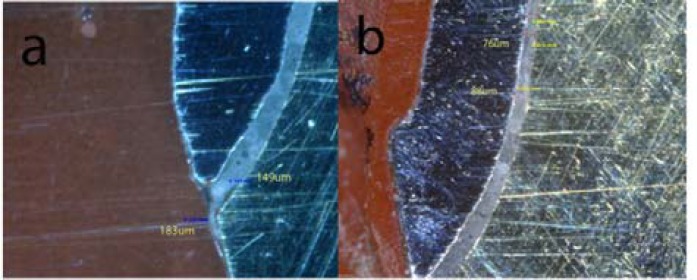
Digital images representitive of absolute marginal discrepancy (AMD=183um), marginal gap (MG=149um) (a) and internal gap axial(IGA=88um) (b) of CAD/CAM group

**Figure 11:**
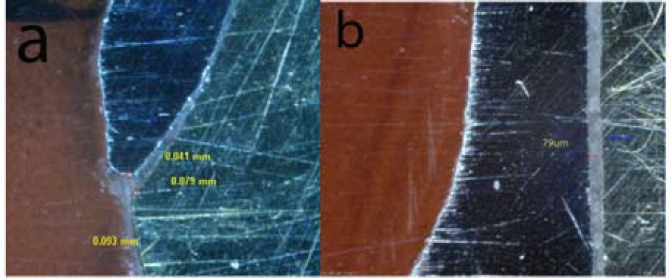
Digital images representitive of absolute marginal discrepancy (AMD=93um), marginal gap(MG=79um) (a) and internal gap axial(IGA=79um) (b) of conventional group

The Student’s t-test indicated that marginal and internal adaptation was significantly different among the 2 groups. The CAD/CAM group had significantly larger gaps at all measured areas than conventional group (*p*< 0.001), especially the AMD and MG, which were 254.45 ±25.09 and 157.37±20.63 for CAD/CAM group and; 88.08±10.66 and 69.54±15.60 for conventional Group respectively. Images of representative specimens from the CAD/CAM and conventional groups are shown in Figures 10 and 11 respectively.

## Discussion

The aim of this study was to compare the marginal and internal fit of metal copings cast from machine-milled wax patterns and conventionally hand-made wax patterns.

The results support the rejection of the null hypothesis; as there was a significant difference in the marginal and internal gap between the two methods of fabrication. The fit of conventional group is better in all measured area than CAD/CAM group (Table I).

Most investigators continue to use the criteria established by McLean and von Fraunhofer [31] for evaluating a clinically acceptable maximum marginal gap width. Considering this only the amount of marginal discrepancies of conventional group are within the clinically acceptable range of 120um. But Moldovan et al. [40] rated the values of 100 um for marginal misfit as good and values of 200–300 um as acceptable. Therefore, the marginal accuracies, represented by the absolute marginal discrepancy and measured in the present study, could be rated as good for conventional group and as acceptable for CAD/CAM group.

The most important criterion dentists use to evaluate the clinical acceptability of cast restorations is their marginal fit [41]. The internal fit is a clinically relevant topic and can affect the strength of a crown-cement system. Lack of adequate fit is potentially detrimental to both the tooth and the supporting periodontal tissues due to the cement solubility or plaque retention [39, 24]. 

The reference points for measurements and the descriptive terminology defining fit, vary considerably among investigators. Often the same term is used to refer to different measurements, or different terms are used to refer to the same measurement. The terminology described by Holmes et al [39] was used to measure the marginal and internal gap in the present study. The absolute marginal discrepancy and marginal gap is chosen for measuring the marginal discrepancy because the latter is most critical due to cement solubility and the former is always the largest measurement of error at the margin and would reflect the total misfit at that point (vertically and horizontally) [39].

In the cross-sectional technique used in the present study, the samples must be sacrificed and also it is more time-consuming [6, 34]. However, it has greater precision in measurements; as measurement points are more accurate and repeatable. It allows determination of horizontal marginal fidelity (over contouring) and as crown is cemented to die, avoids potential damage from repeated use of master die. Therefore this method was chosen for measuring the marginal and internal fit.

In clinical practice, natural teeth show a large variation because of their age and individual structure, thus causing difficulties in getting standardized abutments. Therefore, in this study, standardized brass dies were used for measuring the marginal and internal fit.

To normalize the measurement, all of the procedure for both groups were standardized except wax patterns fabrication, for instance standardized brass dies were used, almost equal cement space of 30um were applied, uniform thickness and shape of the copings were ensured by master wax-up for both groups, exactly the same investing and casting procedure were accomplished, static load for cementation were used, fixing all the specimen in the same direction at an exact straight line mesio-distally and occluso-cervically, cutting all the copings-dies assemblies exactly at the same plane and determining the exact axial points for measurement by three straight, fine lines.

The die spacer on the conventional group dies were cleaned with steam cleaner after wax pattern fabrication to provide the cement space approximately 30um equal to CAD/CAM group.

Using water jet cutter in this study had several advantages, for instance it has the ability to cut material without interfering with its inherent structure, as there is no “heat-affected zone” (HAZ). Minimizing the effects of heat allows metals to be cut without harming or changing intrinsic properties [42]. It also have several edge quality, which if the higher numbers are used, the surface would be smoother, that reduce or omit the time required for polishing the surfaces as it has little or no saw-induced metal flash or smearing found in routine cutting method [38]. It also reduces the total time required for cutting the specimen by one single-computer controlled cut. 

The milling machine (CORiTEC 340i, imes-icore GmbH) used in this study had four milling axis. Beuer et al. [43] reported that the quality of the restoration does not necessarily increase with the number of processing axes. The quality results much more from the result of the digitalization, data processing and production process.

Although CAD/CAM systems have the potential to enhance accuracy by omitting several conventional fabrication methods, they have introduced some additional steps to the fabrication process that may result in inaccuracies, namely scanning, software design, milling and material processing [6]. Beuer et al. [43] reported that besides the sintering process; scanning procedure, the processing of the geometric data collected, the calculation of milling parameters, and the actual milling process are factors that affect the fitting accuracy of zirconia restorations.

The quality of the 3-D image of a tooth preparation is responsible for the internal and marginal adaptation of the final machined restoration [7]. So the additional steps of scanning and software limitations could partially explain the larger marginal and internal gap found in the CAD/CAM group compared to conventional group. The scanning systems have the limitation of finite resolution; which can result in edges that are slightly rounded and leads to interfering contacts at the incisal/occlusal edges [6]. Reich et al. [21] also reported that systems; which depend on optical impression, experience problems with rounded edges and positive error (which simulates virtual peaks near the edges, so-called ‘over-shooters’). The ‘rounded edges’ and ‘over-shooters’ phenomena have been described for the Cerec intraoral camera [44], but they apply to all CAD/CAM systems that acquire their optical impression by means of striation projection such as the scanner (3Shape D810; 3Shape, Copenhagen K, Denmark) used in current study. Since there is no elevation of the die geometry in reality, an increase of internal discrepancy may result. Also the scan spray that used to inhibit the reflection during scanning could somehow increase the internal gap, as it makes a fine layer on the brass dies.

The results in this study was in agreement with previous studies [9, 14, 21-24], which all reported the prominence of conventional method of metal copings fabrication than CAD/CAM fabricated restoration, due to marginal and/or internal fit. Han et al. [24] and Tan et al. [9] both reported that conventional wax-up and casting technique produced better marginal fit than CAD/CAM technique for fabricating titanium crowns. Valderrama et al. [22] reported the overall marginal discrepancies of 61±34µm for titanium metal ceramic crowns fabricated with CAD/CAM system and 47±17µm for the gold platinum-palladium metal ceramic crowns fabricated with conventional methods; although there were no statistical differences between the two groups. 

It has been demonstrated that marginal fit is significantly dependent on the CAD/CAM system used [37]. There is one similar aspect between all of these studies, that they use subtractive method to fabricate the restorations, out of an industrially prefabricated solid block of ceramic or titanium.

The CAM technologies can be divided into three groups according to the technique used:

1. Subtractive technique from a solid block.
2. Additive technique by applying material on a die (a combination of additive and subtractive CAM approaches)
3. Additive technique by using solid free form (SFF) fabrication or rapid prototyping (RP) [7-8].

Subtractive method of manufacturing has some limitations, as the precision fit of the inside contour of the restoration depends on the size of the smallest usable tool for each material of a system and if the cutting tool was larger in diameter than some parts of the tooth preparation, the CAM system face a dilemma of cutting or not cutting the parts, that result in decrease of internal fit precision or inferior marginal properties, respectively [7, 21]. As found by some authors, there is a reverse relation between marginal and internal fit of CAD/CAM systems used [15, 24, 38, 45]. A large internal gap width have smaller marginal gap dimensions and smaller axial gap could contribute to an under seating restoration and larger occlusal and marginal gap because of the binding of the restoration and the die. Also, most of the cutting tools are incapable of cutting sharp internal angles; which results in an internal binding and consequently an open margin [7].

To avoid this problem, a spacer parameter of the CAD/CAM system had to be chosen, or the fit of the crown had to be corrected by the technician, with a handpiece, during the laboratory try-on procedure. Both procedures might induce wider internal gaps [21]. Beuer et al. [46] reported that the provision of internal relief in the process called ‘radius cutter’, which adds about 50um space at the chamfer area, is expected to result in better seating at the margin. Bindle and Mormann [47] found greater internal space for the DCS, resulted in less marginal gap; which can lead to less premature contact internally. Internal space of 50um used by Gonzalo et al. provided a high precision of fit of restorations [21]. Similarly larger cement space was considered for CAD/CAM or CAM groups in several studies [9, 14-15]. It is obvious that, though this increased cement space decreases the marginal gap, it may lead to critically large internal gaps that may adversely affect the mechanical properties of the cements [14, 21]. However, due to the ductility of the metal core, the internal fit is not an important topic for determination of the success of the metal-ceramic core [33].

This study did not support the results reported by some authors [25-28]. Although it is difficult to compare the results between these studies, but there is a similarity due to the CAD/CAM systems used in these studies. Almost all of these studies used Procera (Procera; Noble Biocare, Goteburg, Sweden) [25-26, 28] or DCS (DCS Dental AG; Allschwil, Switzerland) [27] systems to fabricate either titanium or zirconium crowns or copings. It has been demonstrated that adaptation primarily depended on the type of the computer-aided manufacturing (CAM) system [14]. There are some differences between digitization systems and CAM techniques used by these two systems. Both systems use mechanical scanner to detect and record a surface [7]. Previous studies have demonstrated more precision in mechanical digitalizing than optical scanning [25, 47-48]. The problems regarding the digitization of edge-shaped surfaces and angled areas of tooth preparations have been described when using optical scanners [21, 29, 48-49]. Also, Procera system (Procera; Noble Biocare) uses a combination of additive and subtractive CAM approaches for fabrication of inner and outer surfaces of aluminum oxide copings respectively and a combination of spark erosion for the inner contour and milling for the outside contour of titanium frameworks [7]. These findings could partly explain the better results for Procera system than other CAD/CAM systems and conventional methods, in the literature. As reported by Martiez-Rus et al. [29] fewer laboratory steps and the precision of both the digitizing method and the industrial fabrication process for the Procera system resulted in better marginal fit comparing to other systems that had evaluated in the study.

 But when CAM techniques of subtraction from a solid block and additive application on a die, compared with regard to their fit to the die, both techniques yield good results after manual precision adjustments by a dental technician [50].

The mean marginal and internal gap of conventional group was within the range of the values reported by previous studies [33, 51-52], and the mild increase in the values reported in this study might be because of the cementation procedure or using base metal alloys for casting as it has been proven that cementation increase [12, 41, 53-54] and using high noble alloys decrease the marginal gap [1, 55]. Also, most of them had measured the vertical marginal discrepancy that is always smaller than absolute marginal discrepancy used in this study. 

In the current study, a recognized common feature was a significantly greater occlusal gap (IGO) than the axial one (IGA). This is in agreement with previous studies [24, 56]. The occlusal gap ranges between 100-160um in size [57] that is almost consistent with values in the current study. In the present study, the adaptation of copings was assessed without porcelain veneering because the copings principally define the overall adaptation of veneered crowns [35, 58].

There are some limitations in this study. Marginal fits of copings before cementation were not determined. Moreover, cemented copings were not subjected to thermal cycling. Thermal cycling is one of the important factors that affect the long-term marginal fit of crown [41]. Also all coping were produced and tested under ideal conditions, which may not reflect conditions in daily clinical practice. Standardized brass dies were used for measuring marginal and internal fit; however the use of human teeth would be the ideal for simulating a clinical procedure.

Suggestions for future studies include marginal and internal fit evaluation of copings cast from CAD/ CAM fabricated wax patterns following varying amounts of simulated die spacer to determine if there was an optimal amount of simulated die spacer. Also by introducing rapid prototyping techniques, it is possible to overcome all of the subtractive method short comings. Future studies in both fields are needed to find a better alternative for conventional methods of metal coping fabrication.

## Conclusion

Within the limitations of this study and the material and methods used, the following conclusions may be drawn:

1. The conventional method of wax-patterns fabrication resulted in better marginal and internal fit of final copings than CAD/CAM milled wax-patterns at all measured area.

2. The marginal and internal fit of copings cast from CAD/CAM milled wax-patterns were clinically unacceptable.

3. Although CAD/CAM technology has already changed dentistry, it needs some improvement in scanning procedure, data processing, manufacturing techniques and material processing to be a competitive alternative for conventional method of fabrications.
